# Leveraging electronic medical records for HIV testing, care, and treatment programming in Kenya—the national data warehouse project

**DOI:** 10.1186/s12911-023-02265-6

**Published:** 2023-09-15

**Authors:** Margaret Ndisha, Amin S. Hassan, Faith Ngari, Evans Munene, Mary Gikura, Koske Kimutai, Kennedy Muthoka, Lisa Amai Murie, Herman Tolentino, Jacob Odhiambo, Pascal Mwele, Lydia Odero, Kate Mbaire, Gonza Omoro, Davies O. Kimanga

**Affiliations:** 1 Division for Global HIV & TB (DGHT), Centres for Global Health, US Centres for Disease Control and Prevention (CDC), P. O. Box 606, Nairobi, 00621 Kenya; 2 KEMRI/Wellcome Trust Research Programme, Kilifi, Kenya; 3 National AIDS and STI Control Programme (NASCOP), Ministry of Health, Nairobi, Kenya; 4 Palladium Group, Nairobi, Kenya; 5 Division for Global HIV & TB (DGHT), Centres for Global Health, US Centres for Disease Control and Prevention (CDC), Atlanta, Georgia; 6 United States Agency for International Development (USAID), Nairobi, Kenya; 7 PEPFAR Coordinating Office (PCO), Nairobi, Kenya; 8 US Department of Defence (DOD), Nairobi, Kenya

**Keywords:** Electronic medical records, Repository, Data warehouse, HIV, Surveillance

## Abstract

**Background:**

Aggregate electronic data repositories and population-level cross-sectional surveys play a critical role in HIV programme monitoring and surveillance for data-driven decision-making. However, these data sources have inherent limitations including inability to respond to public health priorities in real-time and to longitudinally follow up clients for ascertainment of long-term outcomes. Electronic medical records (EMRs) have tremendous potential to bridge these gaps when harnessed into a centralised data repository. We describe the evolution of EMRs and the development of a centralised national data warehouse (NDW) repository. Further, we describe the distribution and representativeness of data from the NDW and explore its potential for population-level surveillance of HIV testing, care and treatment in Kenya.

**Main body:**

Health information systems in Kenya have evolved from simple paper records to web-based EMRs with features that support data transmission to the NDW. The NDW design includes four layers: data warehouse application programming interface (DWAPI), central staging, integration service, and data visualization application. The number of health facilities uploading individual-level data to the NDW increased from 666 in 2016 to 1,516 in 2020, covering 41 of 47 counties in Kenya. By the end of 2020, the NDW hosted longitudinal data from 1,928,458 individuals ever started on antiretroviral therapy (ART). In 2020, there were 936,869 individuals who were active on ART in the NDW, compared to 1,219,276 individuals on ART reported in the aggregate-level Kenya Health Information System (KHIS), suggesting 77% coverage. The proportional distribution of individuals on ART by counties in the NDW was consistent with that from KHIS, suggesting representativeness and generalizability at the population level.

**Conclusion:**

The NDW presents opportunities for individual-level HIV programme monitoring and surveillance because of its longitudinal design and its ability to respond to public health priorities in real-time. A comparison with estimates from KHIS demonstrates that the NDW has high coverage and that the data maybe representative and generalizable at the population-level. The NDW is therefore a unique and complementary resource for HIV programme monitoring and surveillance with potential to strengthen timely data driven decision-making towards HIV epidemic control in Kenya.

**Database link:**

(https://dwh.nascop.org/).

## Background

The HIV pandemic remains a public health challenge four decades since the first documented case. In 2020, there were an estimated 1.5 million new HIV infections and 680,000 HIV-related deaths [1]. Significant strides have been made in the fight against the pandemic including a reduction in the number of new HIV infections and HIV-related mortality by 52% and 64% since their peaks in 1997 and 2004, respectively [1]. Whilst evidence from research and modelling studies cautiously point towards the feasibility of achieving epidemic control by the year 2030 [2], translating research interventions into programme policies remains arduous [3, 4], and robust surveillance of HIV programme activities for timely and data-driven decision-making remains a challenge.

Historically, paper medical records (PMRs) were the primary data source for HIV programme monitoring and surveillance in Kenya. Whilst practical, PMRs had inherent challenges, including prescription and transcription errors, leading to data quality concerns. Other PMR-related challenges include tedious reporting processes, duplication of tasks, and lack of robust audit trails [5]. These challenges rendered PMRs less effective for patient management, reporting, and timely decision-making.

Over the last decade, Kenya adopted the District Health Information System (DHIS) for aggregate-level indicator reporting purposes [6]. The DHIS is an open-source web-based data platform that has revolutionised aggregate-level data reporting, making it a rich resource for real-time disease surveillance and mapping of outbreaks. The major limitation with aggregate-level data repositories, including the DHIS, is their inability to provide granular data at the individual-level for a robust analysis of the burden, distribution, and risk factors of prevalent and emerging public health challenges for targeted interventions.

Because of the challenges with PMRs and aggregate-level electronic data repositories, Kenya also conducts periodic population-based surveys. These include the Kenya demographic health surveys (KDHS) [7–9], Kenya AIDS indicator surveys (KAIS) [10, 11] and, more recently, Kenya population-based HIV impact assessments (KENPHIA) [12]. These cross-sectional stratified random sampling surveys are a unique source of representative and well-characterised individual-level data. They have proved invaluable in providing an evidence-base for decision-making and policy formulation. However, these surveys also have inherent limitations. First, the cross-sectional design implies that the surveys cannot be used to elucidate longitudinal outcomes. Longitudinal surveillance is increasingly relevant in HIV programming as the pandemic has transitioned from an acutely fatal disease to a chronic manageable, and long-term condition [13]. Second, these surveys are done periodically, mostly after every five years, and therefore cannot be leveraged to respond to new and emerging HIV programme priorities in real-time for decision-making.

In addition to population-level surveys, Kenya has also leveraged technology to design electronic stand-alone data platforms for monitoring programme-specific indicators like HIV RNA viral load and early infant diagnosis databases [14, 15]. These platforms offer rich sources of individual-level data, albeit indicator-specific, that are mostly used to monitor point estimates and temporal trends. The primary limitation with these indicator-specific stand-alone repositories is their inability to delineate the HIV care and treatment continuum for a holistic approach to HIV programme surveillance.

With technological advances, many health facilities in Kenya have adopted electronic medical records (EMR) for patient management. If harnessed into a centralised repository, EMR data have immense potential to enhance HIV programme monitoring and surveillance. In recent years, the country embarked on a project aimed at leveraging EMR to develop a national-level data repository for the HIV programme referred to as the national EMR data warehouse (NDW) project. Here, we describe the evolution of EMR systems in Kenya and their contribution to the NDW. Then, we describe the design and features of the NDW. Lastly, we describe the distribution of the PLHIV captured in the NDW and compare with other national level systems to explore its coverage, representativeness and generalisability for HIV programme surveillance in Kenya.

## Content and construction

### Evolution of electronic medical records in Kenya

#### Introduction of HIV/AIDS management guidelines and scale-up of antiretroviral therapy

**Fig. 1:**
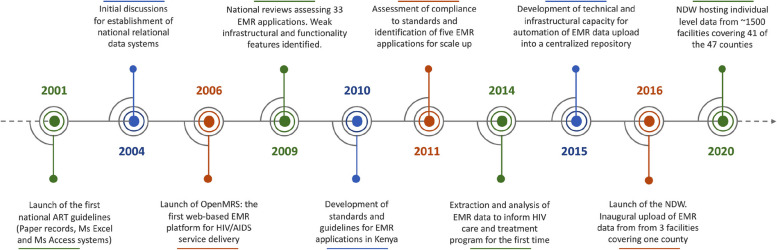
Background and evolution of electronic medical record applications and the national EMR data warehouse (NDW) in the backdrop of ever-evolving technology and HIV program guidelines and policies between 2001 and 2020 in Kenya

In Kenya, EMRs have been in use over many years for various purposes, including patient management, supply chain management, and human resource management. Introduction of EMR for HIV programming, initially in the form of simple windows-based (Ms Excel, Access) platforms, coincided with the launch of the first national guidelines for HIV voluntary counseling and testing in 2001 [16]. The introduction of combination antiretroviral therapy (ART) in 2004 necessitated initial discussions for establishing national electronic data capture systems. Thereafter, EMR were increasingly adopted to improve HIV programme efficiencies in data collection, data management, reporting, and decision-making (Fig. 1).

#### Establishment of electronic medical records standards and guidelines

Between 2007 and 2009, three separate reviews of EMR systems in Kenya were done to assess their design, functionality, and operationalization [17]. Combined, these assessments reviewed 33 EMR systems deployed in 69 facilities in Kenya. Overall, the assessments observed that the systems were developed in the absence of clear guidelines and standards for EMR. Specifically, the assessments identified weak infrastructural frameworks, gaps in functionalities, weak interoperability features and poorly defined operationalization processes. These findings informed the recommendation to establish standards to guide the design, development, deployment, and national scalability of EMR.

In 2010, guidelines for the standardization of EMR were developed with an initial focus on the HIV/AIDS programme [18]. In brief, the guidelines were broken down into three sections: (i) EMR systems development, with pre-requisite processes to be undertaken, minimum essential functional features to be met, and critical software attributes to be included to ensure high-quality data collection, (ii) EMR system deployment, facilitating interaction between-and-within modules, interoperability with other HIS and data transmission (e.g., via Health Level 7 (HL7) and Statistical Data and Metadata eXchange (SDMX) messaging) to a centralised electronic data repository, and (iii) EMR implementation, with best practice requirements for seamless operationalization, oversight, maintenance support, data governance and a continuous review of processes.

In 2011, a review of EMRs was done to assess compliance to established standards and guidelines, provide a framework for technical support towards standardization, and identify high-performance applications for national scalability [19, 20]. The review was carried out in 28 facilities supporting 17 EMR across the country. The applications were assessed and scored against a pre-determined minimum set of features and functionalities as defined in the standards and guidelines document [18]. The systems reviewed were supported by various back-end platforms, including MySQL, MSSQL, MS Access, Oracle, and PostgreSQL. Only 6 systems were web-based. EMR systems with the highest weighted scores were OpenMRS AMPATH (AMRS), IQ Care, C-PAD, and Funsoft. Findings from this review informed the development of a road map for system-specific upgrades to meet established standards and guidelines and scale-up at the national level.

#### Scale up of electronic medical record systems and development of the national data EMR warehouse

In 2014, EMR data were leveraged to inform the HIV programme at the national level for the first time. In brief, data from ~ 500,000 HIV-infected individuals captured in 17 applications deployed in 345 facilities covering 41 of the 47 counties were used to assess twelve-months retention amongst individuals starting ART in Kenya. This inaugural analysis demonstrated the unique potential of EMRs as sources of individual-level data to support decision-making. However, the manual process of data extraction, data collation, and data cleaning underscored the need to automate data transmission from EMR into a centralised repository.

In 2015, the development of technical capacity and physical infrastructure for automating individual-level data transmission from EMR into a centralised repository began. In 2016, the NDW was officially launched. The inaugural upload was done in May 2016 and comprised data from two EMR applications deployed in three facilities covering one county (Meru). By the end of 2020, the NDW was hosting individual-level longitudinal data from five EMR applications deployed in ~ 1,500 facilities (out of the 3,458 [44%] ART sites), covering 41 of the 47 counties in Kenya (Fig. 1).

### The national data EMR warehouse

#### Sources of data

The NDW is a centralised electronic data repository of routinely collected individual-level HIV programme data transmitted from EMR deployed in health facilities offering HIV testing, care, and treatment services in Kenya. Data is collected from clients and either captured in case report forms (CRFs) for retrospective data entry or entered directly into EMR via mobile devices or desktops, or a hybrid of both. CRFs include the HIV Testing Services form (HTS form, or MOH 362) [21], and the clinical encounter services form (also called the green card, or MOH257) [22]. Based on variables from CRFs, a comprehensive data dictionary was developed and informed the database schema in the NDW. Then, the data warehouse application programming interface (DWAPI) was installed and configured alongside the EMR at the health facility server to facilitate data transmission to the NDW. DWAPI facilitates vertical interoperability by allowing disparate EMR systems to all transmit data in similar formats into the NDW. However, there are currently no feature to facilitate horizontal (between EMRs) interoperability. Data transmission is done routinely at the end of every month.

#### Architecture and design

**Fig. 2:**
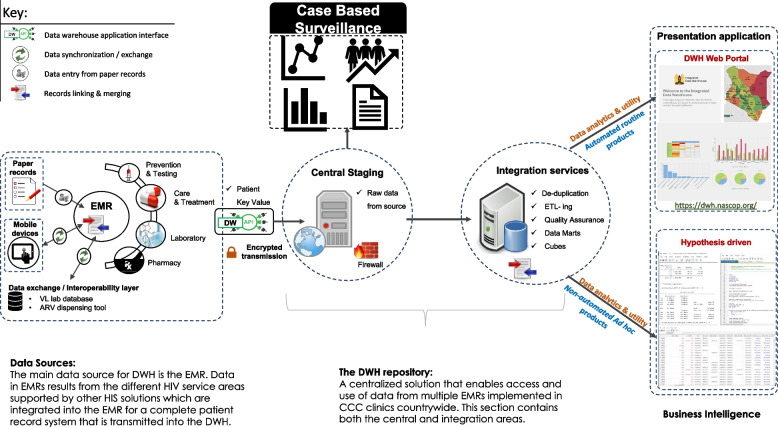
Architecture and design of the national EMR data warehouse (NDW) outlining the five components of the repository and illustrating infrastructure, processes, and function involved in each component, through from individual-level data collection to data utility at the national level

The NDW is comprised of four layers, including DWAPI, the central staging, the integration service, and the data visualization application layers (Fig. 2). DWAPI enables secure data transmission from multiple sources to a centralised repository through the Secure Sockets Layer (SSL). DWAPI’s core architecture is developed using Angular and .NET Core frameworks and supports both MSSQL and MySQL environments. Before uploading, data are processed for de-duplication, de-identification, and generation of a patient key value (PKV) identifier. In brief, a PKV is generated using patient demographics (<gender><soundex(fname)><double metaphone(lname)><dob(yyyymmdd). Probabilistic matching is also undertaken where an index record is further compared with other variables (name, date of birth, gender, date of enrolment, etc.) for matches, and a Jaro-Winkler distance score is calculated on the PKV [23]. Those that score a match of > 95% are considered as possible matches. DWAPI also generates data quality logs for system administrators to synthesize, and detailed feedback is reverted to users at facilities for corrective measures.

The central staging layer consists of encrypted API endpoints that use REST protocols to receive data over the internet in JSON format. This layer temporarily stores raw data as received from DWAPI and supports MSSQL, MySQL and Postgres environments. Due to high volumes of data received from multiple EMRs simultaneously, data are queued using features from RabbitMQ, MSMQ, and Hangfire for asynchronous background processing services. Data from all sources are merged into one dataset. An extract, transform and load (ETL) pipeline is then applied to extract and process data into separate data marts based on different data transformation cleanup routines.

The integration service layer comprises high-capacity data servers that store processed data marts. Processed data marts are organized and integrated into the servers using the integration service layer. When needed for analytics, relational data models are applied to extract and process data marts, which are subsequently transmitted for different use cases, including the generation of routine automated or non-routine ad hoc informational data products. By the end of 2020, the server’s mainframe was running on a Windows Server 2019 with both MySQL version 8 and MS SQL 2017 database platforms with an Intel Xeon 2.2 GHz Central Processing Unit (CPU).

The data visualization application layer receives processed data marts from the servers and applies them for data visualizations using Highcharts, an interactive open-source data visualization software. Highcharts dashboards are developed and configured to allow for interaction, analysis, visualization, and exportation of data outputs based on pre-defined indicators. Informational products are made accessible on the web-front end of the NDW servers using Java Scripts for easy access by stakeholders and the public. This layer also runs customized SQL queries for download of line-listed datasets to be used for in-depth hypothesis driven data analyses.

The NDW continues to evolve in response to changing HIV programme guidelines and to keep abreast with technological advancements towards enhancing its functionalities for efficient, seamless, and secure data transmission, data archiving, and generation of interactive informational products.

#### Data quality

Data quality concerns in the NDW project are inherent and consistent with any repository leveraging routine program data. Addressing data quality challenges in the NDW is an on-going priority. Data quality is ensured through established structures and policies that are routinely evaluated and upgraded to conform to the evolving NDW environment. In brief, these include: (i) policies governing training for data entry, transmission, management and archiving, (ii) systemic structures embedded within EMR applications including quality checks for mandatory fields, null values, missing values, range checks, logic validations and data completeness, and (iii) Institutionalization of routine data quality assessment (RDQA), data review meetings and feedback structures for EMR end-users, mid-level managers and NDW administrators.

#### Data use

##### Routine automated data products

The NDW is used to generate routine automated informational data products. These are broadly classified into process indicator reports, quality monitoring reports and HIV programme performance reports. Process indicator reports are generated monthly and include indicators like proportion of facilities uploading data and timely upload of EMR data. Quality monitoring reports are generated monthly and include indicators like proportion of facilities with erroneous data entries and missing data. HIV programme performance reports are auto-generated on-the-fly to provide real-time updates on national performance indicators of priority including numbers tested for HIV (and positivity yield), numbers started and current on ART and numbers tested for HIV viral load (and virologic suppression). All automated reports are configured, and the results visualized using high-charts dashboards on the publicly accessible NDW web interface.

##### Non-routine ad hoc data products

Data from the repository is also leveraged to respond to non-routine and emerging HIV/AIDS programme priorities for timely decision-making. Individual level data is extracted from the repository and an analytic approach is applied to respond to specific questions and hypotheses. Data analysis is carried out using statistical software like R, Stata or SPSS, and results presented in the form of power-point slide sets, technical reports, bulletins, policy briefs, and peer-reviewed manuscripts to counties, national and global HIV programme teams.

#### Data safety, security, and backup

The NDW uses a 256-bit encryption to ensure that data are secured during transmission. A secure socket layer (SSL) has also been integrated to enable a secure connection between the DWAPI client and the API (server). A Sophos XG firewall and VPNs limit access to and secure the warehouse from the public internet. All EMR and the NDW have role-based access controls and password restrictions, with different cadres of staff having different levels of authorization depending on the organization units.

The NDW is configured to run on two separate (primary and secondary) sites located away from each other and are secured from fire and theft. Data in the warehouse is backed up weekly to an off-site backup server and an external drive kept in a secure location. The server rooms are kept in air-conditioned locked rooms accessible only to registered persons with biometric credentials and server room access keys. The main frame of the NDW and all the servers are only accessible remotely via secure FORTI-CLIENT and SOPHOS VPNs.

#### Data governance and ethical considerations

The NDW project is establishing data governance structures comprising a steering committee and a policy document. The mandate of the data governance steering committee is to oversee the development, implementation, and compliance with data governance policies and regulations. The data governance steering committee will be comprised of stakeholders from the Ministry of Health, the National HIV/AIDS and STI Control Programme, US government agencies, service delivery partners and patient representative groups. Data governance policy and regulations are under development to outline a framework that will uphold privacy and confidentiality, enhance data protection and security, ensure compliance with data regulations, and inform data access, sharing and data use.

Since data are collected from routine service delivery and are intended to be used for national HIV programme evaluation, clients have not been asked for their informed consent. Instead, ethics approval to collect, archive and use data from the repository without retrospectively seeking informed consent from clients, has been obtained under a non-routine non-research determination (NRD) protocol (P716/2019) from AMREF’s Health Africa Ethics and Scientific Review Committee (ESRC). This project was reviewed in accordance with CDC human research protection procedures and was determined to be research, but CDC investigators did not interact with human subjects or have access to identifiable data or specimens for research purposes (CDC, reference number 2018–528).

## Utility

### Scale up of data upload and enhancement of features over time

**Fig. 3:**
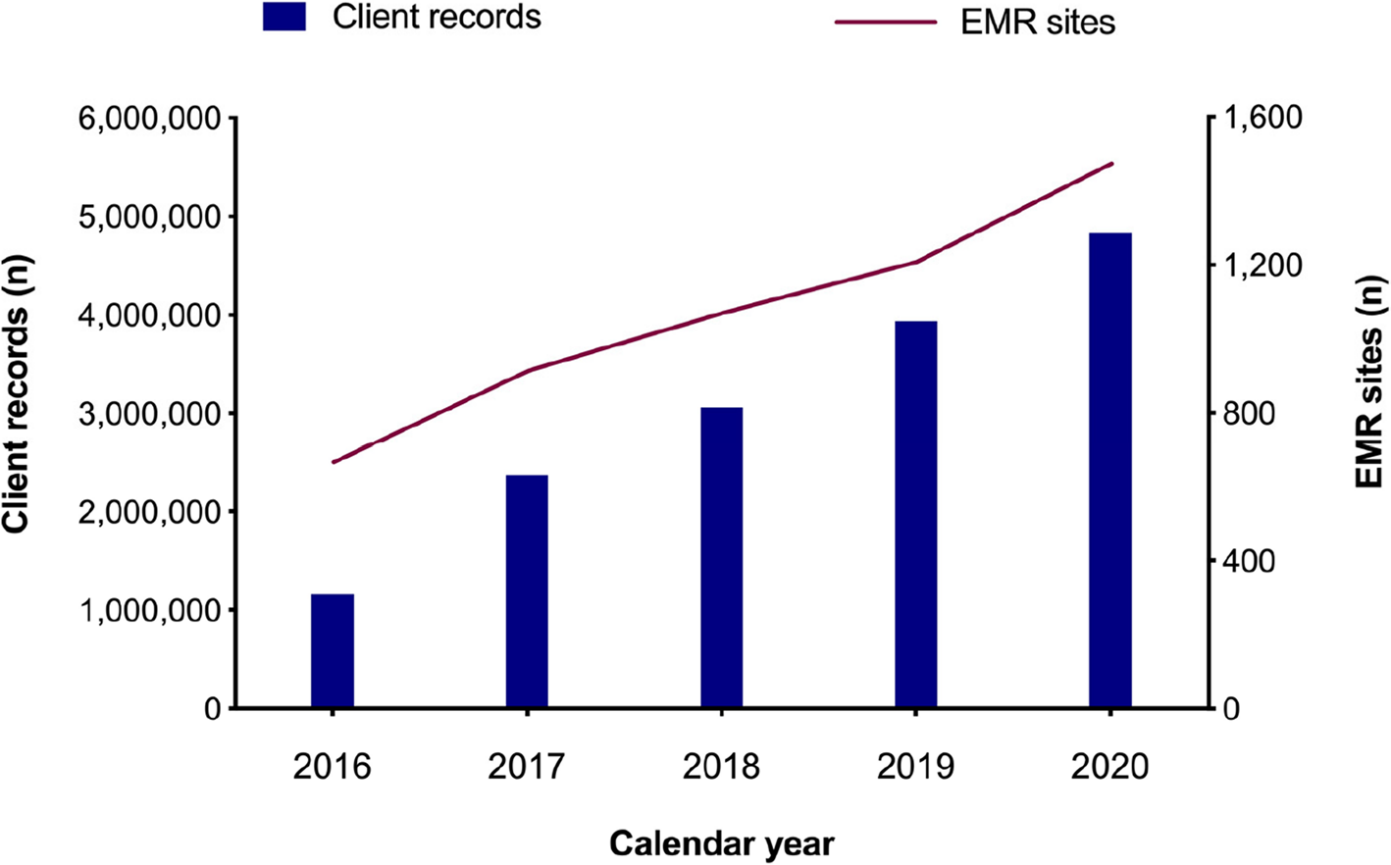
Graph showing number of EMR sites uploading data into the NDW and the number of client records uploaded onto the National EMR Data Warehouse (NDW) between 2016 and 2020

The inaugural upload of data to the NDW was May 2016. By the end of 2016, an estimated 666 facilities had uploaded data from a cumulative 1,162,778 individuals onto the repository. These included individuals seeking HIV testing services and those enrolled for HIV care and treatment services. By the end of 2020, an estimated 1,516 facilities had uploaded data from a cumulative 4,833,786 individuals onto the repository (Fig. 3).

Over time, major enhancements were also implemented on the NDW infrastructure and functionalities in tandem, in response to the large upload volumes, and to keep abreast with technological advancements (Table 1). Plans are underway to enhance the NDW to include data marts and corresponding reports and dashboard for priority HIV program areas including prevention of mother to child transmission (PMTCT), pre-exposure prophylaxis (PreP), key populations (KP) and voluntary medical male circumcision (VMMC).

**Table 1:** Enhancements implemented in the NDW infrastructure and functionalities to respond to the increasing number of sites and volumes uploaded in the repository between 2016 and 2020

Enhancements	2016	2017	2018	2019	2020
Functionalities of the NDW	DWH Portal developed GIS visualization Total dashboards (5)	Transfer Hosting of DWH to NASCOP Servers Resources page developed Total dashboards (32) Basic Upload verification portal (SPOT) developed	DWH Logins disabled Training materials, Dictionaries, Reports uploaded to Resources page Total dashboards (49) SPOT Updated for program (HTS & C&T) upload verification—Different Instances Data Mart Growth (104.1 GB)	Hosting at NASCOP infrastructure for Primary services to increase ownership and adoption of resource Training & engagement of MOH for ownership Migration to Opensource dashboarding tool (HighCharts) to align with Principles of digital development SPOT enhanced as a single instance for all dockets (C&T, Prevention and PKV) Data Mart Growth (488 GB) Enhanced security of DWH data Access & Transmission processes (DWAPI, DWH and other related APIs)	Secondary Infrastructure moved to a Tier II data center to increase availability and security DTG and Palantir data extraction APIs Migration of Home page, Reporting Rates, HIV Testing and Care and Treatment Dashboards to Highcharts SPOT tool enhanced for metrics review and data upload status verification checks to increase data verification process Data Mart Growth (709.5 GB) Enhanced Security and availability of the DWH infrastructure through acquiring DWH hosting services
Functionalities on DWAPI	(Data Upload Application-DeUAP). The tool was used to upload CSV based files that were downloaded from EMRs to the warehouse	First version of DWAPI was developed as a desktop software application Windows-based (running on Microsoft.NET Version 4.6) system that allowed transmission of Care and Treatment data only Supported database connection to KenyaEMR and IQCare EMR software systems only Other EMRs required to generate CSV file extracts to be imported into DWAPI	Migrated from windows-based desktop software application to a web-based platform Expanded support for other EMR software systems, eliminating the need for CSV file extracts Source code migrated from a Microsoft Windows-focused framework to a cross platform.NET Core 2.0 framework that supported running on Linux and MacOS Operating System environments in addition to the natively supported Microsoft Windows platform Deduplication feature was added to support facility patient matching and removal of duplicate patient records	Enhanced to collect and transmit HIV Testing Services and Linkage to care data in addition to wider Care and Treatment data coverage	Enhanced to support service delivery partners with single installation of EMR software collecting and transmitting data from multiple facilities Enhanced to support a community model setup where facilities are supported by more than one partner offering different modalities of HTS services

### Distribution of the HIV care and treatment surveillance population

By the end of 2020, the NDW hosted individual-level longitudinal data from 1,928,458 clients ever started on antiretroviral therapy (ART) in Kenya. These data were transmitted from six EMR applications deployed by 35 service delivery partners supporting 1,516 out of 3,458 (44%) facilities offering care and treatment services, covering 41 of the 47 counties in Kenya (Table 2).

**Table 2:** A summary of service delivery partners supporting electronic medical record applications that have ever transmitted individual-level data from antiretroviral treatment facilities to the National EMR Data Warehouse (NDW) by the end of 2020 in Kenya

Service Delivery Partner	EMR applications supported	Counties supported	Facilities supported	Total clients
Afya jijini	Kenya EMR, IQCare	Embu, Meru, Nairobi, Nyandarua, Tharaka-Nithi	110	110,855
Afya kamilisha	Kenya EMR	Embu	1	547
Afya pwani	Kenya EMR, IQCare	Kilifi, Kwale, Lamu, Mombasa, Taita Taveta	79	78,299
Afya ziwani	Kenya EMR	Kisumu, Nyamira	66	35,699
AHF kenya	Kenya EMR	Makueni, Mombasa, Nairobi	6	10,881
Ampath plus	Kenya EMR, AMRS	Bungoma, Busia, Elgeyo-Marakwet, Homa Bay, Kakamega, Kisumu, Nandi, Trans Nzoia, Turkana, Uasin Gishu, Vihiga, West Pokot	201	376,395
Amref nairobi kitui	Kenya EMR	Nairobi	8	26,364
Aphia plus imarisha	IQCare	Isiolo, Marsabit, Tana River	13	2,326
Bomu hospital sites	Kenya EMR	Kilifi, Kwale, Mombasa	6	24,751
CHAK CHAP uzima	Kenya EMR	Embu, Isiolo, Kajiado, Kiambu, Kilifi, Kirinyaga, Kitui, Laikipia, Machakos, Makueni, Meru, Mombasa, Muranga, Nairobi, Nakuru, Narok, Nywandarua, Nyeri, Taita Taveta, Tharaka Nithi	82	86,502
CHS naishi	Kenya EMR, IQCare	Kitui, Machakos, Makueni	105	95,217
CHS shinda	Kenya EMR	Siaya	119	139,283
CHS tegemeza plus	Kenya EMR	Muranga, Nyandarua, Nyeri	68	66,414
COGRI	Kenya EMR	Nairobi	8	3,560
Columbia stars	Kenya EMR	Kisumu	1	10,576
Coptic hospitals	Kenya EMR	Kisumu, Nairobi	5	21,494
EDARP	ECare	Nairobi	14	61,864
EGPAF timiza	Kenya EMR	Homa Bay	157	143,968
HJF-Kisumu west	Kenya EMR	Kisumu	7	9,016
HJF-Nairobi	Kenya EMR	Mombasa, Nairobi	2	1,980
HJF-south rift valley	Kenya EMR, IQCare	Bomet, Kericho, Nandi, Narok	72	52,883
HOPE worldwide Kenya	Kenya EMR	Embu, Kirinyaga, Nyeri, Tharaka-Nithi	4	702
IRDO	Kenya EMR	Kisumu	1	426
KCCB KARP	Kenya EMR	Bungoma, Busia, Homa Bay, Kakamega, Kisii, Kisumu, Migori, Siaya, Vihiga	56	127,440
LVCT daraja	Kenya EMR, IQCare	Kiambu, Nairobi	8	8,113
LVCT prisons	Kenya EMR, IQCare	Embu, Kajiado, Kakamega, Kericho, Kilifi, Kisii, Kisumu, Machakos, Mombasa, Murang’a, Nairobi, Nakuru, Nyeri, Taita Taveta, Tana River	24	7,778
LVCT steps	Kenya EMR	Kisii, Kisumu, Migori	11	7,581
Ngima for sure	Kenya EMR	Siaya	6	6,241
UCSF clinical Kisumu	Kenya EMR, OpenMRS	Kisumu	24	60,632
UMB PACT endeleza	Kenya EMR	Nairobi	32	43,766
UMB timiza	Kenya EMR	Kisii, Migori	64	87,014
UON COE niche	Kenya EMR	Nairobi	3	29,047
UON CRISSP plus	Kenya EMR	Kiambu, Kirinyaga, Muranga	45	63,939
USAID tujenge jamii	Kenya EMR	Baringo, Kajiado, Laikipia, Machakos, Nakuru, Samburu	81	103,196
No partner	Others	Kajiado, Kericho, Kiambu, Kilifi, Kisumu, Kitui, Meru, Migori, Mombasa, Nairobi, Nakuru, Nyeri, Siaya, Taita Taveta, Uasin Gishu	27	23,709
**Totals**			1,516	1,928,458

**Fig. 4:**
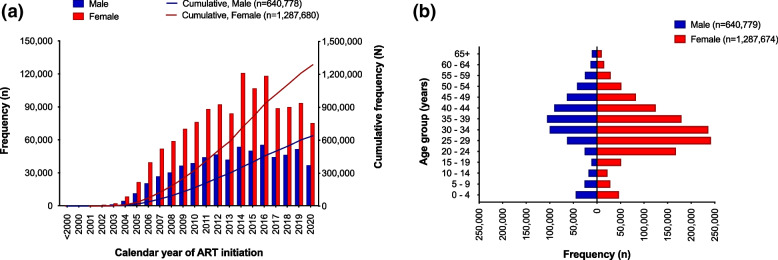
**a** Graph illustrating absolute and cumulative frequency of clients starting antiretroviral treatment by gender over calendar years, and **b** Population pyramid showing distribution of clients by gender and age groups, from facilities transmitting individual level data from electronic medical record applications to the national EMR data warehouse (NDW) in Kenya (*N* = 1,928,458)

Overall, the NDW hosts longitudinal data from individuals who started ART dating back to the early 2000s. Individuals starting ART increased exponentially from the year 2004. Two peaks in the number of individuals starting ART were observed around 2010–2011 and 2014–2016 (Fig. 4a). Majority of clients starting ART were female (*n* = 1,287,674 [66.8%]), and this was consistent over the years. The median age at ART initiation among children (< 15 years) was 5.1 (IQR, 1.8 – 9.4) years, with the majority starting ART at < 5 years. Among adults, male clients were older at ART initiation compared to females (median age, 38.4 [IQR, 32.0 – 46.1] vs. 32.6 [IQR, 26.7 – 40.4] years, respectively). The highest proportion of male clients starting ART were 35–39 years (*n* = 105,517 [16.5%]) whilst that of female clients was 25–29 years (*n* = 241,836 [18.8%]) (Fig. 4b).

### Comparing the spatial distribution of the NDW surveillance population to other data sources

**Fig. 5:**
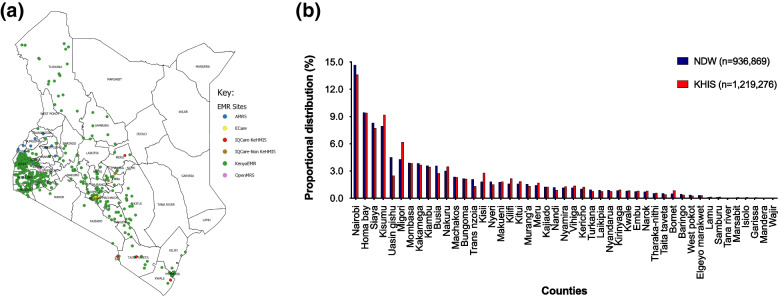
**a** Map showing geospatial distribution of electronic medical record (EMR) applications that transmitted individual level data to the national EMR data warehouse by EMR type in 2020 (*produced by the author using QGIS and the data used for this analysis; the author owns the copyright and can reproduce it*), and **b** proportional distribution of individual on antiretroviral therapy and in active care in 2020 by counties, using data from the National EMR Data Warehouse (NDW) (*n* = 936,869) and the Kenya HIS (*n* = 1,219,276)

Indicators from the Kenya Health information Systems (KHIS), a well-established national aggregate-level data repository, were used. The number of individuals on ART and on follow-up care in 2020 was used as a proxy indicator for comparison with data from the NDW. From the NDW, an estimated 936,869 individuals picked an ART drug re-fill at least once in 2020. Drugs were picked from facilities supported by the six EMR applications deployed in 41 counties. Most of the facilities were using the KenyaEMR system. Most of the facilities are clustered around the western part of the country (Fig. 5a).

Overall, the proportional distribution of individuals by counties from the NDW was consistent with that from KHIS (Fig. 5b). In brief, data from both the NDW and KHIS demonstrated that Nairobi County contributed the highest proportion of individuals on ART in 2020. Indeed, Nairobi (14.1% vs 13.6%), Siaya (9.8% vs 7.7%), Homa bay (9.6% vs 9.4%) and Kisumu (9.3 vs 9.2%%) were the top four counties with the highest proportional contribution of individuals on ART from both the NDW and KHIS, respectively. Further and by 2020, there were no EMRs deployed in six counties in the north-eastern part of Kenya, including Wajir, Mandera, Garissa, Marsabit, Isiolo and Lamu, because of their very low to negligible burden of HIV. Similarly, data from KHIS reported that Wajir County had the lowest proportional contribution (0.02%), followed by Mandera (0.06%), Garissa (0.09%), Marsabit (0.10%), Isiolo (0.11%) and Lamu (0.14%).

## Discussion

Stand-alone electronic data platforms, aggregate-reporting data repositories, and population-level cross-sectional surveys play a critical role in disease surveillance and data-driven decision-making in the HIV/AIDS programme. However, these data sources have inherent limitations in situations requiring real-time public health responses and in longitudinal follow-up of clients diagnosed with HIV. When integrated into the NDW, EMR data have tremendous potential to bridge this gap by providing a longitudinal data platform for tracking HIV programme performance at the patient level facilitating real-time evidence-based decision-making.

Covering 41 of 47 counties, the NDW has extensive geographic coverage. Six counties are yet to be supported to contribute data to the NDW. These are HIV low-burden counties with an estimated zero number of new HIV infections in 2018 [24]. Most of the facilities using EMR systems were concentrated in counties around the Lake Victoria in Western Kenya, particularly Homa Bay, Siaya, and Kisumu. This is a reflection of the high HIV burden, with prevalence estimates of up to 19% in the region, and is consistent with the National HIV/AIDS programme strategy of targeted interventions for maximum impact [24]. Importantly and when compared with data from the KHIS as a proxy, the proportional distribution of counties were consistent in ranking and comparable with those from the NDW [24]. In 2020, the NDW captured visits from 936,869 clients on ART, compared to an estimated 1,219,276 clients on ART reported by KHIS, suggesting a 77% representation of PLHIVs on ART in Kenya at the time. Thus, while the NDW has low EMR coverage (1,516 out of 3,483 ART-providing facilities in Kenya [44%]), it seems to achieve high (77%) individual level coverage at the population-level. This observation also suggests that the ~ 56% of facilities not included in the NDW are likely from low volume facilities that capture < 25% of PLHIV on ART in Kenya. These results indicate notable progress towards representativeness and generalisability of the NDW at the population-level.

There was an exponential increase in clients starting ART from 2004, with two subsequent peaks in 2010–2011 and 2014–2016. In 2004, only clients who had WHO clinical staging III/IV or CD4 T-cell count of < 200 cells/mm^3^ were eligible for ART initiation [25]. The CD4 classification was revised over time to CD4 < 350 cells/mm^3^ in 2010, and for all HIV-infected clients regardless of the clinical or immunological status in 2014 [26, 27]. The peaks in ART initiation around these periods reflect the changing guidelines and demonstrate the unique potential for the NDW as a resource to assess impact of changes in guidelines and policies on the HIV programme. Currently, the NDW is the only programmatic resource that offers this unique perspective on the HIV programme in Kenya.

The NDW has two main strengths. First, the longitudinal structure makes it a unique and incomparable resource for ascertaining longitudinal outcomes at the patient-level in Kenya. Second, the monthly data transmission to the NDW makes it possible to respond to new and emerging HIV programme priorities in real-time for decision-making. However, the NDW is not without limitations. First, while tremendous effort has been put towards setting up structures and policies for generation and archiving of high-quality data in the NDW, data quality concerns cannot be entirely ruled out. Second, while the national programme recommends consistent use of the comprehensive care clinic (CCC) unique identifier, compliance continues to present a challenge. To mitigate this, DWAPI applies a de-duplication algorithm that generates a secondary PKV at the source before data are uploaded, which has been shown to yield up to 96% of unique probabilistic matches [23]. Still, mismatches cannot be ruled out. Last, while there is notable progress in the DWH coverage, the remaining sites, which are likely low volume sites, may cater to a different population with unique challenges. This may lead to selection bias and a lack of generalizability and therefore solutions to collect data from non-EMR sites should be explored.

## Conclusions

Tremendous gains have been made by leveraging technological advancements for monitoring and surveillance of the HIV programme in Kenya. Over the last two decades, medical information systems have significantly evolved from simple PMRs to sophisticated web-based EMRs with APIs that support data transmission to a centralised repository. The NDW is a powerful resource with capabilities to monitor longitudinal outcomes and to respond to new and emerging public health priorities in real time. A comparison with estimates from KHIS demonstrates that the NDW has high coverage and that the data maybe representative and generalizable at the population-level. The NDW is, therefore a unique and complementary resource for HIV programme monitoring and surveillance with potential to strengthen timely data driven decision-making towards HIV epidemic control in Kenya.

## Data Availability

All data and materials are housed at the National data warehouse. Upon reasonable request, de-identified data from the National EMR data warehouse, which is managed by the Ministry of Health in Kenya, can be made available. All data requests are to be channeled to the corresponding author, Margaret Ndisha, email: odi2@cdc.gov.
